# Chronic Benign Tubular Albuminuria From Compound Heterozygous Variants in *CUBN*: A Case Report

**DOI:** 10.1177/20543581251317016

**Published:** 2025-02-05

**Authors:** Adam Pietrobon, Mark D. Elliott

**Affiliations:** 1 Division of Nephrology, Department of Medicine, The University of British Columbia, Vancouver, BC, Canada; 2 Providence Health Care, Vancouver, BC, Canada

**Keywords:** Cubilin, CUBN, albuminuria, genetic kidney disease, Imerslund-Gräsbeck syndrome

## Abstract

**Rationale:**

Albuminuria is a commonly used parameter for predicting decline in kidney filtration function. Cubilin, encoded by *CUBN*, is a critical protein involved in protein reabsorption in the proximal tubule. Mutations in *CUBN* lead to Imerslund-Gräsbeck syndrome (IGS), a disorder characterized by vitamin B12 deficiency (and consequences related to that) with or without albuminuria. Recent evidence suggests that C-terminal variants in *CUBN* may lead to albuminuria without other features of IGS.

**Presenting concerns of the patient:**

Here, we report a case of a 52-year-old male with chronic, albumin-predominant, subnephrotic range proteinuria since his teenage years, but preserved estimated glomerular filtration rate (eGFR).

**Interventions:**

Neither angiotensin-converting enzyme (ACE) inhibition nor angiotensin Type II (AT-II) receptor blockade reduced his degree of albuminuria.

**Diagnosis:**

Genetic testing identified 3 distinct pathogenic variants in *CUBN* that were confirmed by segregation analysis to be a compound heterozygous mode of inheritance. All variants were downstream of the intrinsic factor-vitamin B12 binding domain of cubilin. The patient had normal vitamin B12 levels and did not exhibit any features of IGS.

**Outcomes:**

Kidney biopsy was not pursued for this patient as diagnostic clarification was achieved by non-invasive genetic testing alone.

**Novel findings:**

This case highlights several important lessons. First, not all albuminuria is made equal, and forms of tubular albuminuria can exist without compromising kidney filtration function. Second, identifying genetic forms of tubular albuminuria is key to avoiding ineffective interventions (eg, ACE inhibition, AT-II receptor blockade, sodium-glucose cotransporter-2 [SGLT2] inhibition) and unnecessary invasive procedures (eg, kidney biopsy). Third, the location of *CUBN* variants dictates phenotypic consequences, with C-terminal variants leading to albuminuria without vitamin B12 deficiency.

## Abrégé

**Motif:**

L’albuminurie est un paramètre couramment utilisé pour prédire le déclin de la fonction de filtration rénale. La cubiline, codée *CUBN*, est une protéine essentielle impliquée dans la réabsorption des protéines dans le tubule proximal. Les mutations de *CUBN* mènent au syndrome d’Imerslund-Gräsbeck (IGS), un trouble caractérisé par une carence en vitamine B12 (et les conséquences qui en découlent) en présence ou non d’albuminurie. Des données récentes suggèrent que des mutations en C-terminal dans la *CUBN* peuvent conduire à une albuminurie sans manifestation des autres aspects du IGS.

**Présentation du cas:**

Nous rapportons le cas d’un homme de 52 ans présentant depuis l’adolescence une protéinurie chronique (à prédominance d’albumine) de type néphrotique, avec préservation du DFGe.

**Interventions:**

L’inhibition de l’ECA et le blocage des récepteurs AT-II n’ont pas permis de réduire le taux d’albuminurie.

**Diagnostic:**

Des tests génétiques ont permis d’identifier trois mutations pathogènes distinctes dans la *CUBN* qui ont par la suite été confirmées par l’analyse de ségrégation comme étant un mode de transmission hétérozygote composite. Toutes les mutations se situaient en aval du domaine intrinsèque de liaison facteur-vitamine B12 de la cubiline. Les taux de vitamine B12 étaient normaux et le patient ne présentait aucune manifestation caractéristique du IGS.

**Résultats:**

La biopsie rénale n’a pas été réalisée, car le diagnostic a pu être clarifié par un test génétique non invasif.

**Nouvelles données:**

Plusieurs leçons importantes peuvent être tirées de ce cas. Premièrement, les albuminuries ne sont pas toutes égales, et certaines formes d’albuminurie tubulaire peuvent exister sans compromettre la fonction de filtration rénale. Deuxièmement, l’identification des formes génétiques d’albuminurie tubulaire est essentielle pour éviter des interventions inefficaces (p. ex., l’inhibition de l’ECA, le blocage des récepteurs AT-II, l’inhibition du SGLT2) et des procédures invasives inutiles (p. ex., biopsie rénale). Troisièmement, l’emplacement des mutations de la *CUBN* dicte les conséquences phénotypiques; les mutations en C-terminal menant à l’albuminurie sans carence en vitamine B12.

## Introduction

Albuminuria is a ubiquitously employed clinical parameter for predicting risk of kidney function decline.^1^ Strategies to reduce albuminuria, such as with angiotensin-converting enzyme (ACE) inhibitors, angiotensin-II receptor blockers (ARB), and sodium-glucose cotransporter-2 (SGLT2) inhibitors prevent progression to end-stage kidney disease on a population level.^2^ While these medications are designed to reduce albuminuria of glomerular etiology, albumin excretion may arise from other origins, such as tubular or urologic.

Cubilin, encoded by the gene *CUBN*, is a critical protein involved in low molecular weight protein reabsorption in the proximal tubule. Along with its binding partners amnionless (*AMN*) and megalin (*LRP2*), cubilin orchestrates receptor-mediated endocytosis, which is the primary mechanism for albumin reabsorption in the proximal tubule.^3^ Cubilin has an additional role in the small intestine and enables vitamin B12 absorption via binding to the intrinsic factor-vitamin B12 (IF-B12) complex.^4^

Variants in the *CUBN* gene are classically described to cause Imerslund-Gräsbeck syndrome (IGS).^5^ This disorder is characterized by vitamin B12 deficiency (and consequences related to that, such as megaloblastic anemia and growth delay) with or without proteinuria.

Recently, a cross-sectional cohort study demonstrated that biallelic variants in *CUBN* downstream of the IF-B12 binding domain (ie, closer to the C-terminus) leads to isolated proteinuria without other features of IGS.^6^ A subsequent cohort study reproduced these findings, while also observing stability in estimated glomerular filtration rate (eGFR) over an average follow-up of 7 years.^7^ It remains an important question whether eGFR stability persists longitudinally and later into adulthood.

## Presenting Concerns

**Figure 1.**
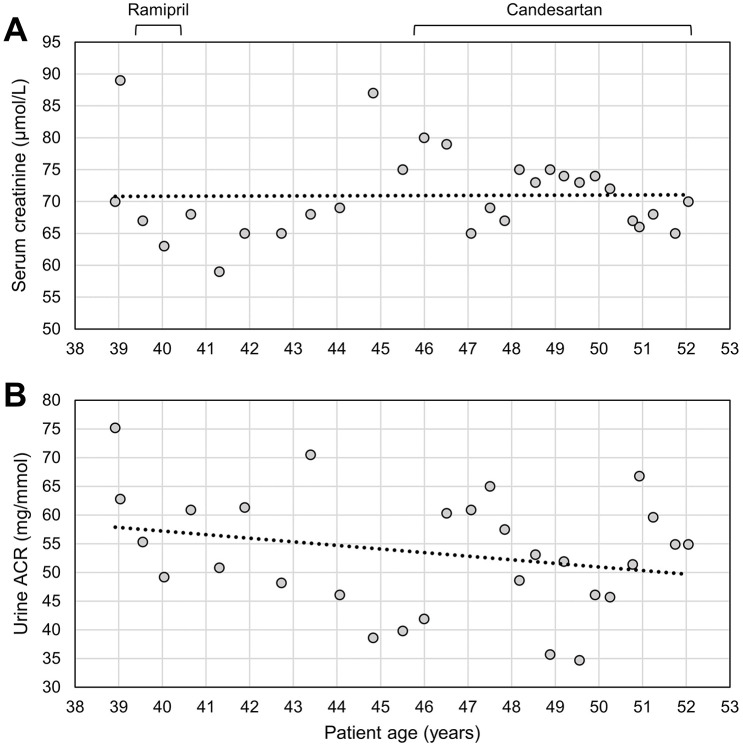
Measured values of patient (A) serum creatinine and (B) urine albumin to creatinine ratio (ACR) across age. Timing of intervention with therapeutics is indicated. Best fit by linear regression is shown with a dotted line.

We present the case of a 52-year-old East Asian male with longstanding albuminuria yet preserved eGFR. He self-reported albuminuria since his late teenage years, although formally our records document albuminuria since the age of 38. His proteinuria is albumin-predominant (50%-60% of his proteinuria) and has remained within the subnephrotic range with urine albumin to creatine ratio of 50 to 100 mg/mmol (Figure 1).

## Clinical Findings

The patient did not smoke and his comorbidities include dyslipidemia, hypertension, and gout, all of which developed in his late 40s. He had a normal serum albumin (47 g/L) and no edema. He was briefly treated with ramipril and demonstrated no appreciable change in his albuminuria; this was discontinued after a few months due to development of an ACE inhibitor-induced cough. He was subsequently trialed on candesartan but discontinued after 1 week due to dizziness and hypotension. Several years later, he developed hypertension and was restarted on candesartan, which has controlled his blood pressure yet has not affected his albuminuria. Over the years, he exhibited intermittent microscopic hematuria (maximum of 1-3 erythrocytes per high power field), without evidence of acanthocytes or erythrocyte casts. Kidney ultrasound and cystoscopy were both normal.

## Timeline

A timeline of his pertinent lab values and interventions are provided in Figure 1.

## Diagnostic Focus and Assessment

**Figure 2.**
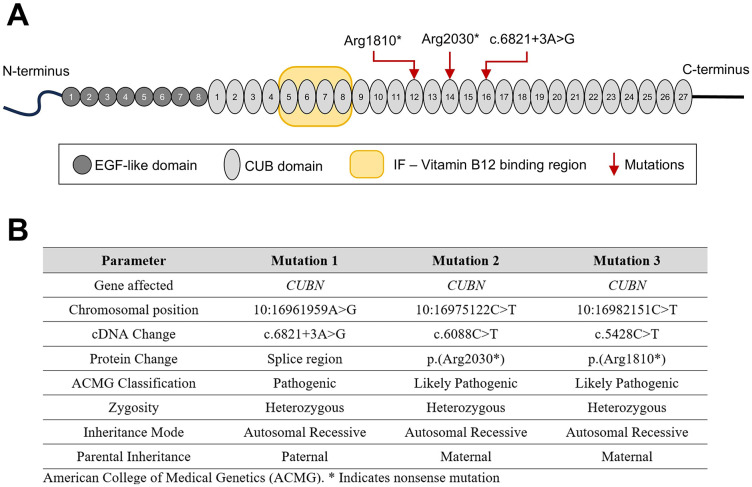
(A) Schematic representation of CUBN protein structure and binding regions, along with location of this patient’s variants. (B) Summary of patient genetic variants. (*) indicates nonsense mutation.

The etiology of his albuminuria remained unexplained for decades. Given the stability of his eGFR and absence of disease progression, a kidney biopsy was never pursued. He was eventually referred to our center for genetic testing. A specimen was analyzed with the Blueprint Nephrotic Syndrome gene panel (Helsinki, Finland, https://blueprintgenetics.com/tests/panels/nephrology/nephrotic-syndrome-panel/), which tests for variants in 96 genes implicated in proteinuria and nephrotic syndrome. The patient was found to have 3 variants in the *CUBN* gene that were deemed pathogenic or likely pathogenic by American College of Medical Genetics and Genomics (ACMG) classification (Figure 2). The first variant, 10:16961959A>G (c.6821+3A>G, splice variant), is a splice variant of likely pathogenicity and was paternally inherited. The other 2 variants, 10:16975122C>T (c.6088C>T, p.Arg2030*) and 10:16982151C>T (c.5428C>T, p.Arg1810*), confer premature stop codons which exist in *cis* and were maternally inherited. Segregation analysis with the unaffected parents confirmed these were germline-inherited variants; of note, his mother possessed 2 variants in *cis*, thus maintaining an unaffected copy of the gene.

Interestingly, all variants were downstream of the IF-B12 binding domain of cubilin (Figure 2). The patient did not have a history of growth delay or congenital anomalies. Furthermore, he had normal vitamin B12 levels (249 pg/mL) and never exhibited evidence of megaloblastic anemia (hemoglobin 157 g/L). Consequentially, he was not diagnosed with IGS.

## Therapeutic Focus and Assessment

After the identification of the *CUBN* variants, the patient was provided with a diagnosis of chronic benign albuminuria. He was advised that he did not require specific treatment with proteinuria-reducing therapy. A kidney biopsy was also not pursued, as his diagnosis was achieved non-invasively via genetic testing. He was advised that his risk of kidney decline most likely mirrors that of the general population.

## Follow-up and Outcomes

The patient continues to be monitored with quarterly lab work. His albuminuria and eGFR remain unchanged. No further invasive work-up as been initiated.

## Discussion

This case report adds to a growing chorus of studies that show biallelic inactivating variants in *CUBN* leads to chronic benign tubular albuminuria without decline in kidney filtration function.^6,7^ We extend previous findings by reporting longitudinal data in an older adult patient, proving stability in eGFR persists with age. It has been previously postulated that proteinuria can confer direct nephrotoxicity via re-uptake of excessive amounts of filtered protein in the proximal tubule.^8^ As our patient cannot effectively resorb protein, the absence of accelerated kidney decline is consistent with such a mechanism. Indeed, in a rat model of adriamycin-induced proteinuria, *CUBN* knockdown reduced tubulointerstitial damage, suggesting a potential protective role cubilin loss.^9^ This case argues that tubular albuminuria does not exhibit the same toxicity profile as glomerular albuminuria.

For this patient, the variants in *CUBN* are downstream of IF-B12 binding domain; consequently, this patient did not exhibit vitamin B12 deficiency or megaloblastic anemia. This report is consistent with prior findings of allelism of the *CUBN* gene, whereby N-terminal variants lead to IGS while C-terminal variants lead to isolated albuminuria.^6^ Of note, our case report rejects the possibility of haploinsufficiency, as both parents remained unaffected while possessing only 1 wildtype allele. Segregation analysis was critical for determining these variants to be in *trans*, which lead to the phenotype observed in our patient.

An interesting finding is the intermittent microscopic hematuria, despite normal cystoscopy and kidney ultrasound imaging. A prior study has also reported a subset of patients with *CUBN* variants exhibit microscopic hematuria.^6^ Mechanistically, it is not clear how *CUBN* variants would confer such a phenotype, as expression is restricted the proximal tubule in the kidney^10^ where no known appreciable exchange of red blood cells occurs. It is possible this hematuria is unrelated to the *CUBN* disease phenotype.

As expected, we show that this tubular albuminuria does not respond to ACE inhibitors or ARBs. Furthermore, the need for kidney biopsy was obviated as a diagnosis was reached using genetic tools alone. This approach highlights the utility of genetic testing early in the diagnostic work-up, thereby avoiding ineffective interventions and unnecessary invasive procedures.
